# Does fetal endoscopic closure of the myelomeningocele prevent loss of neurologic function in spina bifida aperta?

**DOI:** 10.1186/1743-8454-7-S1-S18

**Published:** 2010-12-15

**Authors:** Renate Verbeek, Axel Heep, Natasha Maurits, Reinhold Cremer, Oebele Brouwer, Johannes van der Hoeven, Deborah Sival

**Affiliations:** 1 Department of Neurology University Medical Center Groningen, University of Groningen, Hanzeplein 1, PO Box 30001, 9700RB Groningen, The Netherlands; 2 Department of Neonatology University of Bonn, Sigmund Freud Str. 25, 53105, Bonn, Germany; 3 Department of Pediatrics Children’s Hospital Cologne, Amsterdamer Str. 59, 50735, Cologne, Germany; 4 Department of Pediatrics University Medical Center Groningen, University of Groningen, Hanzeplein 1, PO Box 30001, 9700RB Groningen, The Netherlands

## Background

Spina bifida aperta (SBA) is associated with shunt-dependent hydrocephalus and with meningomyelocele (MMC). Fetal endoscopic closure of the MMC may reduce shunt-dependency, but the benefit upon motor function in individual patients is still unclear. An increase in differentiated muscle ultrasound density (dMUD) provides an objective parameter for the extent of muscle damage caudal to the MMC. In this perspective, we aimed to compare dMUD and neurological function between SBA children treated by fetal endoscopic closure (fSBA) and by neonatal closure (nSBA) of the MMC.

## Materials and methods

We included 12 [age- and (level of) MMC-] matched pairs of fSBA and nSBA children [age 1 (0-5) years; upper level MMC L3 (L2 - L5); medians (ranges)]. All 12 fSBA patients were delivered by caesarean section, all 12 nSBA patients by vaginal delivery. To compensate for the effect by delivery mode, we also compared separate (age- and MMC-) matched pairs of nSBA children born by caesarean section (nSBA-SC; n=13) and by vaginal delivery (nSBA-VD; n=13). Neurological parameters consisted of dMUD (defined as: [MUD caudal to the MMC] minus [MUD cranial to the MMC]); motor- and sensory function and shunt-dependent hydrocephalus and Chiari-II (C-II) malformation. fSBA and nSBA patients were treated at Bonn and Groningen/Cologne, respectively.

## Results

dMUD was significantly lower in fSBA than nSBA [15 (-9 to 68) vs. 26 (-1 to 39), medians (ranges); p<.05]. Assessment of motor and sensory function indicated a lower segmental spinal function in fSBA than nSBA [median difference 2 myotomes and 2 dermatomes (ranges -0.5 to 4 and -1 to 5); p<.05]. Shunt-dependent hydrocephalus appeared less frequent in fSBA than in nSBA (4/12 vs 11/12; p<.05), whereas the incidence of C-II malformation did not significantly differ between fSBA and nSBA (10/12 vs 12/12, respectively). Comparing the neurological parameters between nSBA-SC and nSBA-VD revealed no significant differences.

## Conclusions

Assessment of dMUD and neurological function in fSBA and nSBA children indicates a moderately improved neuromuscular outcome in fSBA. It remains to be established by long-term functional and cognitive outcome parameters to determine whether the suggested neurological benefit is maintained and outweighs the risks of iatrogenic complications by the fetal endoscopic surgery.

